# Supplementary material for: Puf6 primes 60S pre-ribosome nuclear export at low temperature
Source: Nat Commun. 2021 Aug 4;12:4696. doi: 10.1038/s41467-021-24964-2 (PMC8338941; doi:10.1038/s41467-021-24964-2)
Supplement: Supplementary file 1 — Supplementary Information [file 41467_2021_24964_MOESM1_ESM.pdf]

Supplementary Information

Puf6 primes 60S pre-ribosome nuclear export at low temperature

Stefan Gerhardy, Michaela Oborská-Oplová, Ludovic Gillet, Richard Börner, Rob van Nues, Alexander Leitner, Erich Michel, Janusz J. Petkowski, Sander Granneman, Roland K. O. Sigel, Ruedi Aebersold & Vikram Govind Panse

Supplementary Table 1. Yeast strains used in this study

Strain name	Genotype	Origin
BY4741	MATa ura3 his3 leu2 met15 TRP1	Euroscarf
L40 coat	MATa his3-200 ura3-52 leu2-3,112 ade2 LYS2::(lexAop)-HIS3 LexA-MS2 coat (TRP1)	SenGupta *et al.*, 1996 [1]
Puf6-TAP	MATa ura3 leu2 TRP1 PUF6-TAP::HIS3MX	Open Biosystems
Ssf1-TAP	MATa ura3 leu2 TRP1 SSF1-TAP::HIS3MX	Open Biosystems
Ssf1-TAP *puf6Δ*	MATa ura3 leu2 TRP1 SSF1-TAP::HIS3MX puf6Δ::KANMX	this study
Rix1-TAP	MATa ura3 leu2 TRP1 RIX1-TAP::HIS3MX	Open Biosystems
Arx1-TAP	MATa ura3 leu2 TRP1 ARX1-TAP::HIS3MX	Open Biosystems
Kre35-TAP	MATa ura3 leu2 TRP1 KRE35-TAP::HIS3MX	Open Biosystems
Nog2-TAP	MATa ura3 his3 leu2 met15 TRP1 NOG2-TAP::HIS3MX	Open Biosystems
puf6Δ	MATa ura3 his3 leu2 met15 TRP1 puf6Δ::KANMX	this study
bud20Δ	MATa ura3 his3 leu2 met15 TRP1 bud20::KANMX	Altvater *et al.*, 2012 [2]
yrb2Δ	MATa ura3 his3 leu2 met15 TRP1 yrb2::KANMX	Altvater *et al.*, 2014 [3]
Puf6-GFP	MATa ura3 leu2 TRP1 PUF6-GFP::HIS3MX	Open biosystems
Gar1-mCherry Puf6-GFP	MATa GAR1-mCherry:: KANMX ura3 leu2 met15 TRP1 Puf6-GFP:: HIS3MX	this study
kar1-1 NUP82-mCherry	MATalpha ura3-52 leu2-3,112 kar1-1 ade2-101 NUP82-mCherry::KAN	Altvater *et al.*, 2012 [2]
Arx1-GFP	MATa ura3 his3 leu2 met15 TRP1 ARX1-GFP::HIS3MX	Altvater *et al.*, 2012 [2]
Gar1-GFP	MATa ura3 his3 leu2 met15 TRP1 GAR1-GFP::HIS3MX	Altvater *et al.*, 2012 [2]
Nop7-TAP	MATa ura3 his3 leu2 met15 TRP1 NOP7-TAP::HIS3MX	Open biosystems
Nop7-TAP *puf6Δ*	MATa ura3 his3 leu2 met15 TRP1 NOP7-TAP::HIS3MX puf6::NATNT2	this study

Supplementary Table 2. Plasmids used in this study

Plasmid	Features	Source
pRS315-Puf6	PUF6, CEN, LEU2, AMP	this study
pRS315-*puf6* R172E R431E	puf6 R172E R431E, CEN, LEU2, AMP	this study
pRS315-*puf6* Y208E R431E	puf6 Y208E R431E, CEN, LEU2, AMP	this study
pRS316-uL18-GFP	uL18(RpL5)-eGFP, CEN, URA3	Altvater *et al.*, 2012 [2]
pRS316-uS5-GFP	uS5(Rps2)-eGFP, CEN, URA3	Altvater *et al.*, 2012 [2]
pRS315-Pum3	hPUM3, CEN, LEU2, AMP	this study
pET47-Puf6	His_6-Puf6, KAN	this study
pET47-Puf6 (161-656)	His_6-Puf6 (161-656aa), KAN	this study
pEM1-Puf6 (161-656)	His_6-GB1-Puf6 (161-656aa), AMP	this study
pACT2-Puf6	2µ, AMP, LEU2, P_{ADH1}, T_{ADH1}, G4AD-PUF6	this study
p3A-MS2-1	2µ, AMP, URA3, ADE2, P_{PolIII}, MS2 sites, T_{PolIII}	Bassler *et al.*, 2006 [4]
p3A-MS2-25S H22	2µ, AMP, URA3, ADE2, P_{PolIII}, MS2-25S-H22, T_{PolIII}<br>RNA sequence:<br>^{299}GGGTGGTAAATTCCATCT^{316}	this study
p3A-MS2-25S H38	2µ, AMP, URA3, ADE2, P_{PolIII}, MS2-25S-H38, T_{PolIII}<br>Plasmid sequence:<br>^{985}TTTTATGAGGTAAAGCGAATGATTAGAGGT<br>TCCGGGGTCGAAATGACCTTGACCTATTCTCAA<br>ACTTTAAATATGTAAGA^{1064}	this study
p3A-MS2-25S H66	2µ, AMP, URA3, ADE2, P_{PolIII}, MS2-25S-H66, T_{PolIII}<br>Plasmid sequence:<br>^{2150}GCATTGCGATGGTCAGAA<br>AGTGATGTTGACGCAATGT^{2186}	this study
p3A-MS2-25S H68	2µ, AMP, URA3, ADE2, P_{PolIII}, MS2-25S-H68, T_{PolIII}<br>Plasmid sequence:<br>^{2194}GCCCAGTGCTCTGAATGTCAAAGTGAAG<br>AAATTCAACCAAGCGCGGGTAAACGGC^{2248}	this study

p3A-MS2-25S H69	2µ, AMP, URA3, ADE2, P_{PolIII}, MS2-25S-H69, T_{PolIII}<br>Plasmid sequence:<br>^{2249}GGGAGTAACTATGACTCTC^{2267}	this study
p3A-MS2-23S H68 *E. coli*	2µ, AMP, URA3, ADE2, P_{PolIII}, MS2-23S-H68 E. coli, T_{PolIII}<br>Plasmid sequence:<br>^{1835}GCCCGGTGCCGGAAGGTTAATTGATGGGGTTAGCC<br>GCAAGGCGAAGCTCTTGATCGAAGCCCCGGTAAACGGC^{1905}	this study
p3A-MS2-23S $_{Sc}$H68 Tip$_{E. coli}$	2µ, AMP, URA3, ADE2, P_{PolIII}, MS2-25S-H68 S. cerevisiae | 23S-H68 E. coli, T_{PolIII}<br>Plasmid sequence:<br>^{2194}GCCCAGTGCTCTGAATGTCAAAGT^{2217} | ^{1861}GGGTTAGCCGCAAGGCGAAGCTC^{1881}<br>| ^{2228}AACCAAGCGCGGGTAAACGGC^{2248}	this study
p3A-MS2-23S $_{E. coli}$**H68 Tip**$_{Sc}$	2µ, AMP, URA3, ADE2, P_{PolIII}, MS2-23S-H68 E. coli | 25S-H68 S. cerevisiae, T_{PolIII}<br>Plasmid sequence:<br>^{1835}GCCCGGTGCCGGAAGGTTAATTGATG^{1860} | ^{2218}GAAGAAATTC^{2227} | ^{1882}TTGATCGAAGCCCCGGTAAACGGC^{1905}	this study
p3A-MS2-25S H88	2µ, AMP, URA3, ADE2, P_{PolIII}, MS2-25S-H88, T_{PolIII}<br>Plasmid sequence:<br>^{2765}CTTTAGTCCCTCGGAATTTGAGGCTAGAG^{2793}	this study

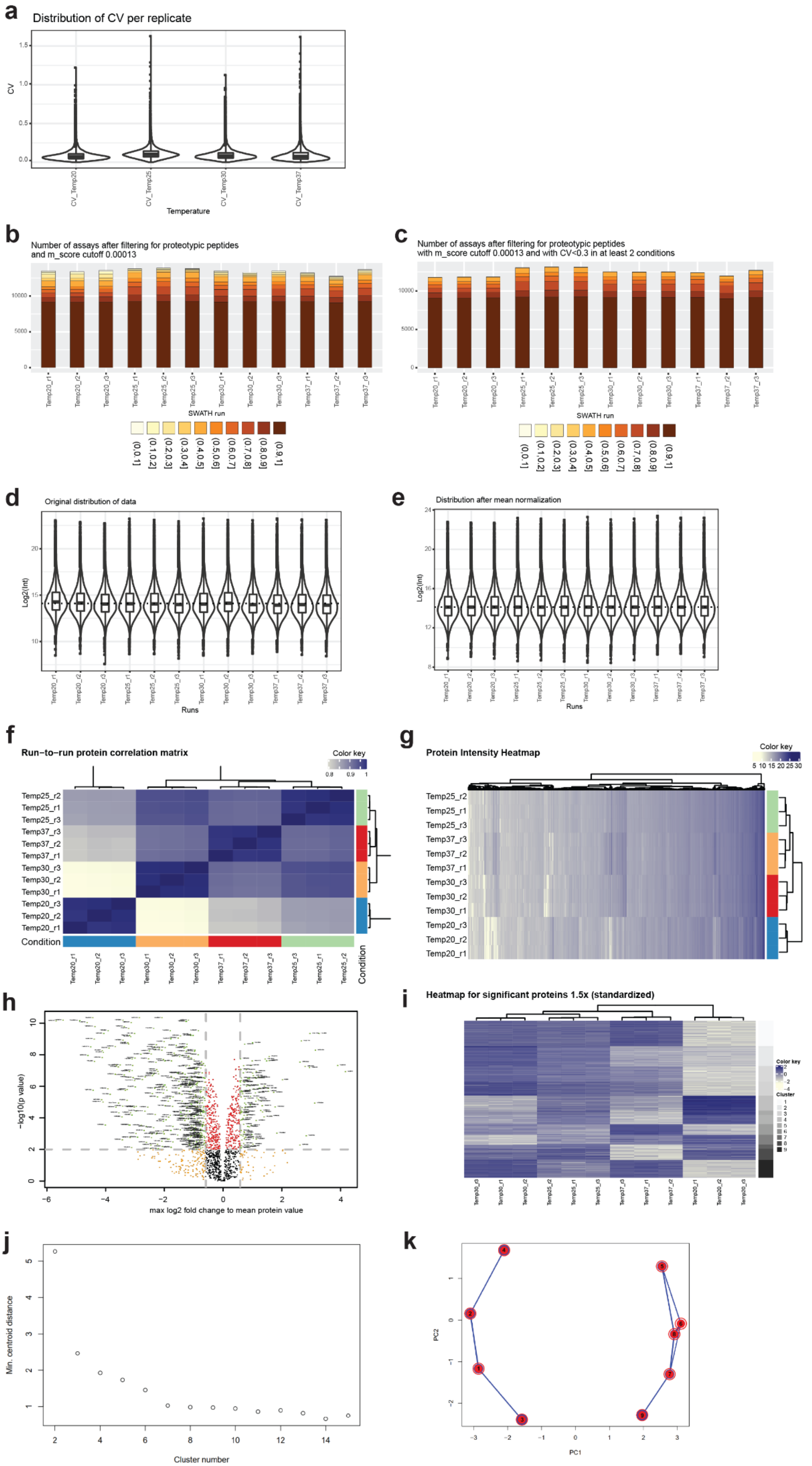
a
Distribution of CV per replicate
CV
Temperature
b
Number of assays after filtering for proteotypic peptides
and m_score cutoff 0.00013
SWATH run
c
Number of assays after filtering for proteotypic peptides
with m_score cutoff 0.00013 and with CV<0.3 in at least 2 conditions
SWATH run
d
Original distribution of data
Log2(Int)
Runs
e
Distribution after mean normalization
Log2(Int)
Runs
f
Run−to−run protein correlation matrix
Color key
Condition
g
Protein Intensity Heatmap
Color key
h
−log10(p value)
max log2 fold change to mean protein value
i
Heatmap for significant proteins 1.5x (standardized)
j
Min. centroid distance
Cluster number
k
PC2
PC1

Supplementary Fig. 1. Supplementary plots of the SWATH MS analysis. All plots were generated from the openSWATH extraction result table, after removing the decoy entries and filtering for 1% protein FDR (corresponding to mScore = 0.00013) and for proteotypic peptides. **a**, Distribution of the coefficient of variances (CV) calculated for the (biological) triplicates per temperature condition. The box plots were constructed using the 25th percentile, 75th percentile and median as the minima, maxima and centre respectively. The upper whisker extends from the hinge to the largest value no further than 1.5 * IQR from the hinge (where IQR is the inter-quartile range, or distance between the first and third quartiles). The lower whisker extends from the hinge to the smallest value at most 1.5 * IQR of the hinge. Data beyond the end of the whiskers are called "outlying" points and are plotted individually. **b**, Bar plot showing the number of peptide assays identified per sample injection and the fraction of times (in 10 bins between [0-0.1] and [0.9-1]) each of those peptide assays were identified across the whole data set. Most of the peptide assays were identified in all runs (category [0.9-1] in brown color). **c**, Same bar plot as for Supplementary Fig. 1b with the additional filtering criteria that the peptide assays should show less than 30% CV across the biological triplicates (see Supplementary Fig. 1a) in at least two temperature conditions. This reduced the total number of assays but increased the proportion of assays seen in all runs (category [0.9-1] in brown color). **d**, Distribution of the Log2 intensity of the identified peptide assays (n = 3 biological independent replicates per condition) before normalization. The box plots were constructed using the 25th percentile, 75th percentile and median as the minima, maxima and centre respectively. The upper whisker extends from the hinge to the largest value no further than 1.5 * IQR from the hinge (where IQR is the inter-quartile range, or distance between the first and third quartiles). The lower whisker extends from the hinge to the smallest value at most 1.5 * IQR of the hinge. Data beyond the end of the whiskers are called "outlying" points and are plotted individually. The dotted line indicates the mean of the means (per run) of the log2 intensities. **e**, Distribution of the Log2 intensity of the identified peptide assays (n = 3 biological independent replicates per condition) after normalizing for the mean of the means. The box plots were constructed using the 25th percentile, 75th percentile and median as the minima, maxima and centre respectively. The upper whisker extends from the hinge to the largest value no further than 1.5 * IQR from the hinge (where IQR is the inter-quartile range, or distance between the first and third quartiles). The lower whisker extends from the hinge to the smallest value at most 1.5 * IQR of the hinge. Data beyond the end of the whiskers are called "outlying" points and are plotted individually. The dotted line indicates the mean of the means (per run) of the log2 intensities. **f**, Non-supervised heatmap of the run-to-run (Pearson) correlation matrix for the intensities of the proteins identified in the whole dataset. **g**, Non-supervised heatmap clustering (Manhattan distance) of the intensities of the proteins identified in the whole dataset. **h**, Volcano plot for the proteins identified in the whole dataset.

Significance of the protein change was assessed using a one-way ANOVA test, with the resulting p-value further adjusted using the FDR method. The x axis represents the maximum log2 fold change to the mean protein value; and the y-axis represents the -log10 of the one-way ANOVA p-value after correction for multiple testing with the FDR method. The proteins with maximum fold change below 1.5 and with a p-value above 0.01 are shown in black. The proteins with maximum fold change above 1.5 and with a p-value above 0.01 are shown in orange. The proteins with maximum fold change below 1.5 and with a p-value below 0.01 are shown in red. The proteins with maximum fold change above 1.5 and with a p-value below 0.01 are shown in green. This last protein category is considered as significantly changing in at least one temperature condition and is annotated with protein names. **i,** Non-supervised heatmap clustering (Manhattan distance) of the standardized intensities of the proteins found significant by the one-way ANOVA test and the 1.5 fold change difference to the mean (proteins labelled in green in Supplementary Fig. 1h). Significance of the protein change was assessed using a one-way ANOVA test, with the resulting p-value further adjusted using the FDR method. The nine most different cluster classes are shown as grey colour coded as colour bar on the left side of the heatmap. **j**, Plot showing the average minimum centroid distance between two cluster centers produced by the c-means clusterings for a given range of cluster number as obtained by the "Dmin" function of the mFuzz package. The stalled decrease in the minimal centroid distance between clusters between the number of clusters 8-10 pointed towards using 9 clusters for the analysis. **k**, Principal Component Analysis plot obtained by the overlap.plot function of the mFuzz package and representing the proximity/similarity of the different clusters. Though the clusters 8 and 6 were quite close/similar, we decided to stick to 9 clusters because the step-wise increase in protein abundance in those two clusters between the temperatures 20°C and 25°C might be of biological interest.

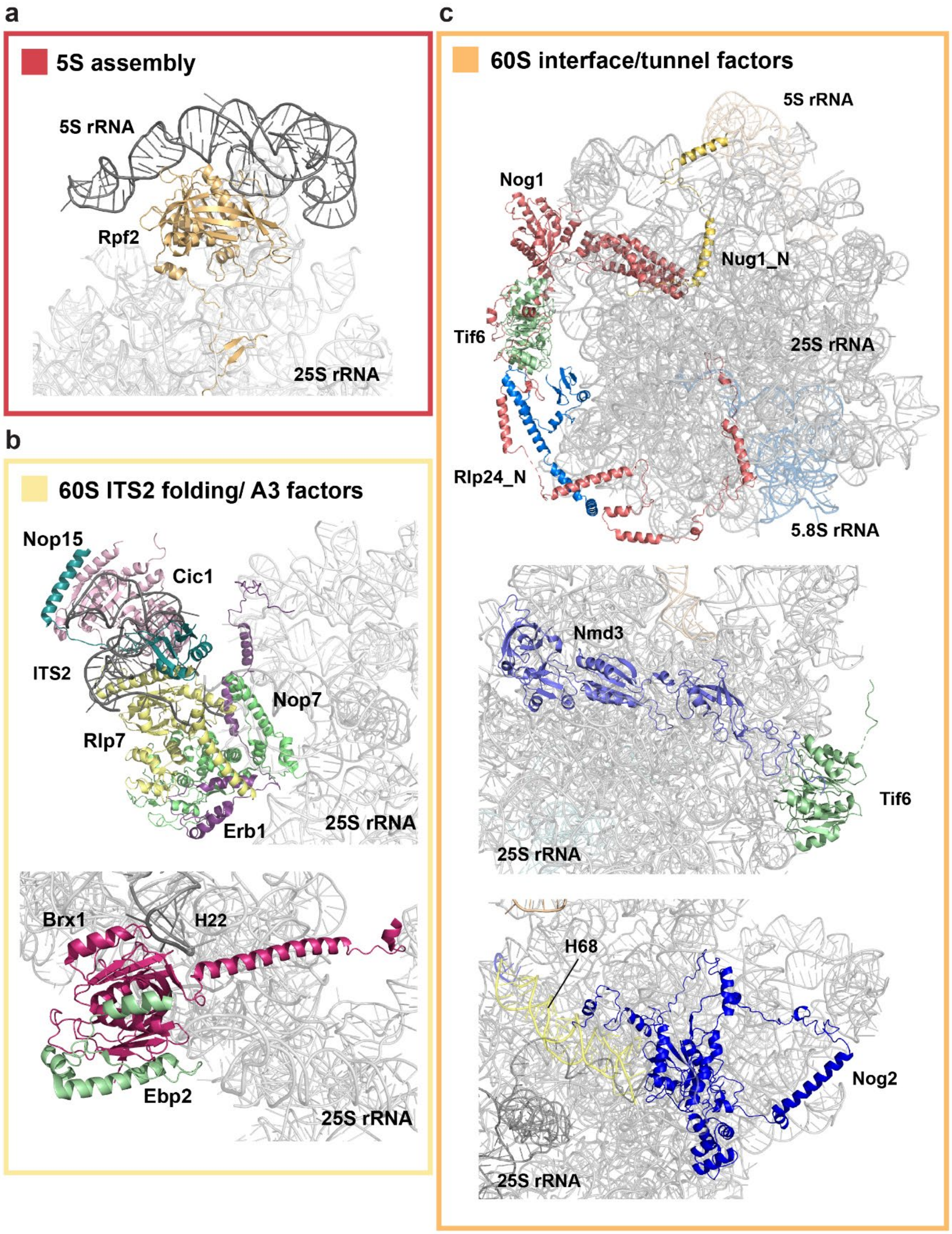

Supplementary Fig. 2. Structural examples of ribosome assembly factors upregulated at low temperature on their RNA-binding sites I. a, Red square representing 5S assembly factors: Rpf2 contacting 5S rRNA and 25S rRNA (PDB ID: 3JCT) [5]. **b**, Yellow square representing A3 factors involved in ITS2 folding and processing. Upper panel: Nop7 in lime, Rlp7 in pale yellow, Nop15 in dark green, Cic1 in pink, Erb1 in purple. Lower panel: Brx1 in hot pink and Ebp2 in green interacting with H22 (PDB ID: 6CB1) [6]. **c**, Orange square

representing factors on the 60S pre-ribosome interface and factors important for the exit tunnel maturation. Upper panel: Nug1_N terminus in yellow, Nog1 in salmon pink inserting its C-terminal tail into the peptide exit tunnel, Rpl24_N terminus in blue intertwined with Nog1 C-terminus, Tif6 in pale green (PDB ID: 3JCT) [5]. Middle panel: Nmd3 in purple spread over the interface of pre-60S covering area from E-site to Tif6 on the other side of the subunit (PDB ID: 6N8M) [7]. Lower panel: Nog2 in blue, located on the inter-subunit surface at the base of H68 (in yellow) (PDB ID: 3JCT) [5]. The figures were generated using PyMoL.

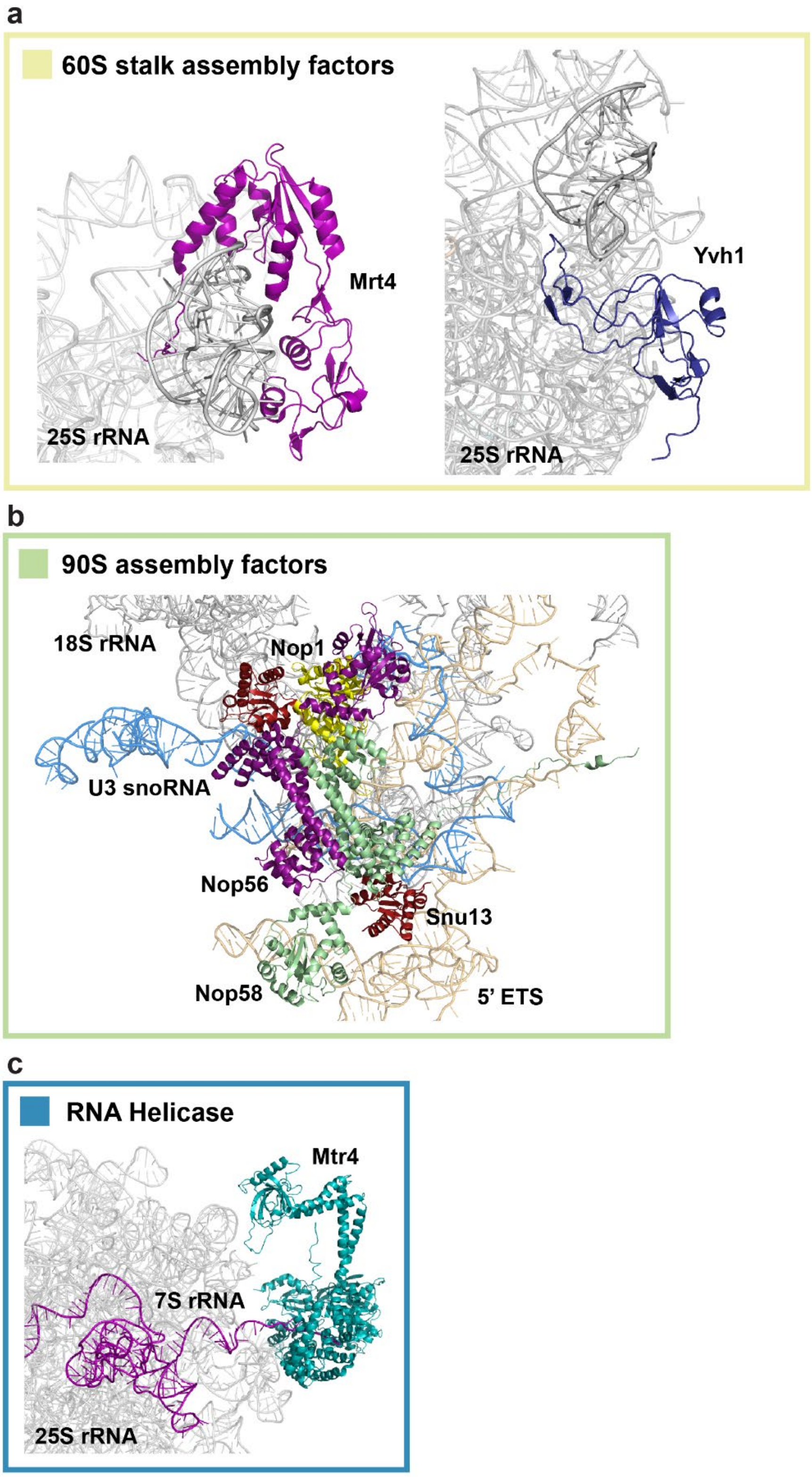

Supplementary Fig. 3. Structural examples of ribosome assembly factors upregulated at low temperature on their RNA-binding sites II. a, Light green square representing 60S stalk assembly factors: Mrt4 in purple (PDB ID: 3JCT) [5], Yvh1 in dark blue which releases Mrt4 [8,9] (PDB ID: 6N8M) [7]. **b**, Green square representing 90S assembly factors, specifically the U3 snoRNP components: Nop56 in purple, Nop58 in green, Snu13 in red and Nop1 in yellow;

interacting with U3 snoRNA in blue and 5’ETS in wheat. (PDB ID: 7AJT) [10]. **c**, Blue square representing RNA helicase: Mtr4 in cyan, which unwinds 7S rRNA and recruits the exosome for 7S rRNA processing (in purple) (PDB ID: 6FT6) [11]. The figures were generated using PyMoL.

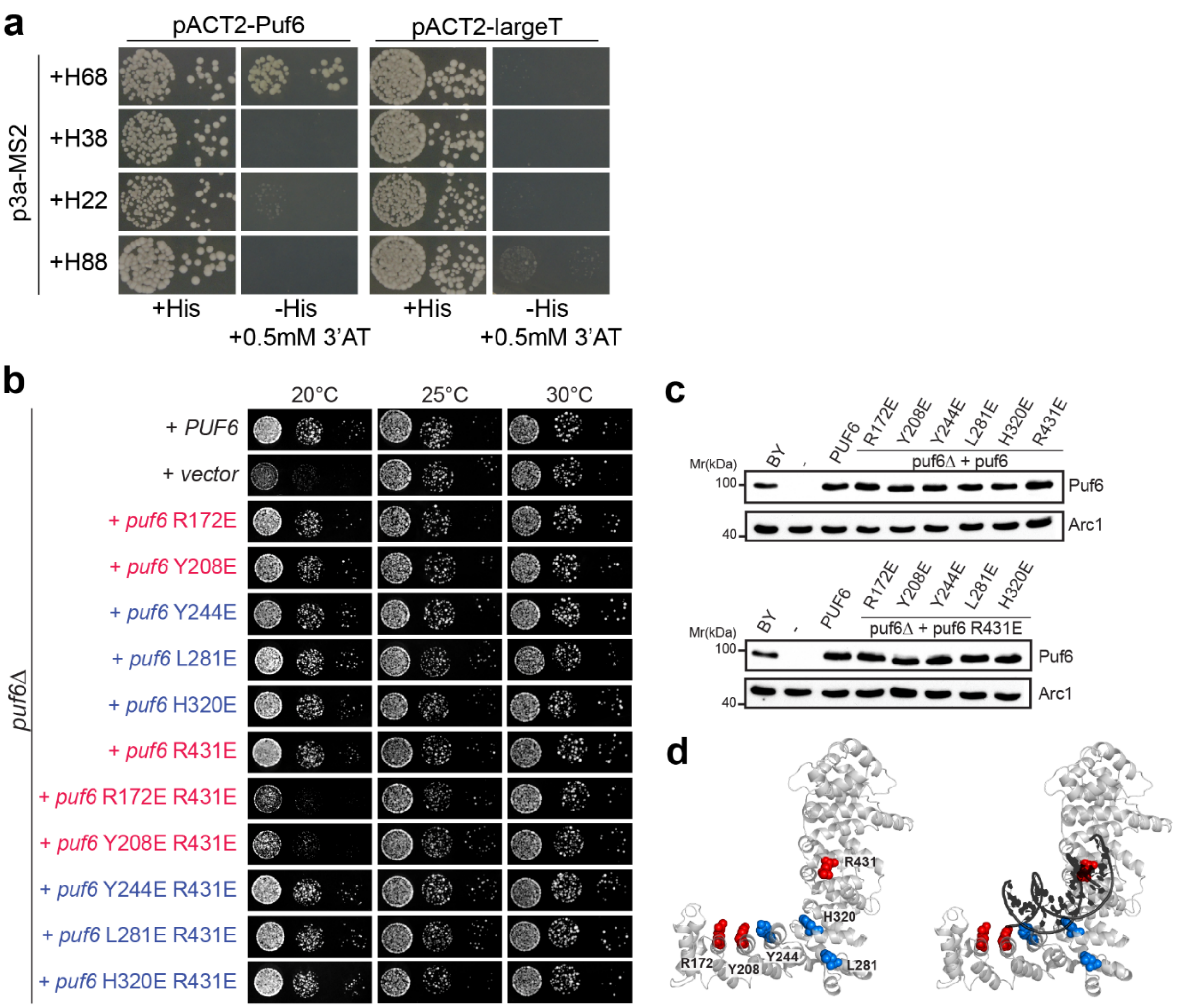

Supplementary Fig. 4. a, Yeast three-hybrid analysis of Puf6 and helices 22 and 88 of 60S ribosomal subunit. L40 strain was co-transformed with p3A-MS2 plasmid expressing indicated helices and pACT2 plasmid expressing Puf6 or largeT (negative control). Transformed cells were spotted in 10-fold serial dilutions on control +His plate and testing -His plate with presence of 0.5 mM 3'AT and grown for 3 days at 30°C. **b**, Single Puf6 amino acid substitutions do not show growth phenotype. Only combined substitutions in the N-terminal patch (R172 and Y208) combined with R431 show a significant growth defect. puf6Δ cells expressing WT-Puf6 or Puf6 single and double amino acid substitutions, were spotted in serial 10-fold dilutions on selective minimal medium plates and grown at indicated temperatures for 3-5 days. Residues in main text in red, additionally tested residues in blue. **c**, Levels of Puf6 single and double mutants are not altered. Whole cell extracts were prepared from puf6Δ cells expressing WT-Puf6 or Puf6 single and double mutants, separated by SDS-PAGE and analyzed by Western blotting with a Puf6 specific antibody. Arc1 was used as a loading control. Source data are provided as a Source Data file. **d**, Puf6 homology model with indicated substituted residues (left) with superimposed dsDNA from Pum3/Puf-A crystal structure (PDB ID: 4WZW) (right). Source data are provided as a Source Data file.

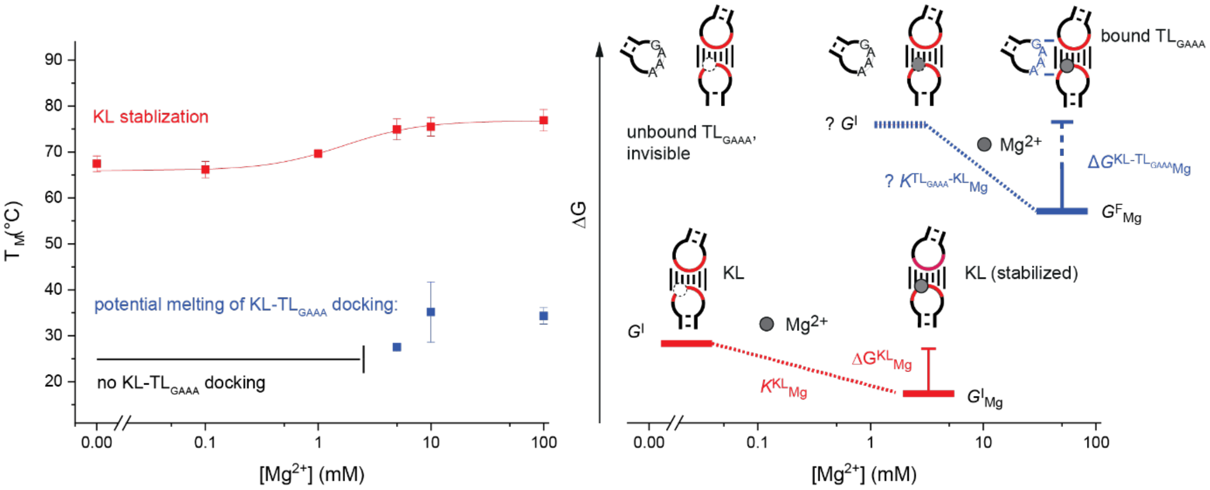

Supplementary Fig. 5. Characterization of KL-TL$_{GAAA}$ RNA construct by RNA UV thermal melting. Left: Mg^{2+} dependent melting temperature of both the kissing loop formation of H22-H88 and the potential TL$_{GAAA}$ of H68 docking to the KL in 20 mM HEPES pH 7.4 buffer containing 116 mM K^{+} and various amounts of Mg^{2+}. The TL$_{GAAA}$ docking is only visible in the presence of > 5 mM Mg^{2+}, in contrast, the KL is present also in absence of Mg^{2+}. Fitting results can be found in Supplementary Table 7. Error bars show mean +/- SD of n = 4 ramps of two individual experiments. Right: Scheme of free energies of the potential Mg^{2+} binding to the formed KL and the subsequent docking of the KL-TL$_{GAAA}$. Source data are provided as a Source Data file.

Chemicals

All chemicals were purchased from Sigma Aldrich of at least puriss grade.

RNA constructs

RNA oligos were purchased from IBA Lifesciences (Göttingen, DE) with cyanine labels at different positions (sCy3/5) to probe particular folding trajectories. Photophysical parameters of cyanine dyes (fluorescence lifetime, time-resolved anisotropy) in the realm of RNA have been previously characterized in detail [12]. Sequences were derived from the 25S ribosomal RNA of wildtype yeast (BY4741) and feature a kissing loop interaction of domains H22 and H88 as well as GAAA tetraloop motif in domain H68 connected via poly-A linker to follow intramolecular folding. Label position of the FRET pair were chosen in such a way to follow both, the GAAA docking of domain H68 to the formed kissing loop and the kissing loop formation of domain H22 and H88, respectively. We refer to these labelled constructs as KL-TL_{GAAA} and kissing loop (KL). Further, we replaced domain H22 and H88, i.e. the kissing loop, with a tetraloop receptor sequence [13] as a control for GAAA tetraloop docking and refer to this construct as tetraloop receptor (TLR-TL_{GAAA}).

All constructs are summarized in Supplementary Table 3.

Knowledge based structure prediction of in vitro construct

Based on the RNA sequences of the *in vitro* construct KL-TL_{GAAA}, a secondary structure prediction was performed with RNAfold leading to based-paired helices, which are in full agreement to the observed helices H22, H68 and H88 in the crystal structure of the 25S ribosomal RNA of wildtype yeast (BY4741). To obtain the kissing loop motif identified between H22 and H88 in the wildtype ribosomal RNA, a knowledge-based pseudoknot motif was introduced (compare Supplementary Table 3). Subsequently, the resulting secondary structure was used to compute a 3D structure prediction with RNAcomposer [14]. This structure indeed shows the KL between H22 and H88. The predicted KL perfectly matches the crystal structure of H22 and H88 within the KL motif, but not the GAAA bound to it. To get the bound conformation of the GAAA containing helix 68 to the KL, we aligned both the predicted KL and helix 68 individually to the wildtype crystal structure minimizing the RMSD and introduced the Poly-A-linker thereafter [15].

RNA sample preparation

RNA constructs were dissolved in ddH_2O and re-buffered in varying amounts of HEPES buffer (0-100mM) pH 7.4 (25°C) established with KOH; potassium(I) (0-100 mM) in 20 mM HEPES, magnesium(II) (0 – 100 mM) in 20 mM HEPES and 100 mM potassium(I) with and without Puf6-PUM (161-656aa) (10 µM) at [RNA] = 1 µM. The actual [K^+] is corrected for the

potassium(I) ions added to adjust the pH 7.4 (25°C) with KOH for different HEPES buffer concentrations according to Henderson-Hasselbalch:

$$[\mathrm{K}^+]_{\mathrm{tot}}=[\mathrm{K}^+]+ [\mathrm{HEPES}]\times 10^{(\mathrm{pH}\text{-}\mathrm{pK_S(HEPES)})}$$

with pK_S(HEPES) = 7.48 (25°C). To allow the RNA to properly fold, RNA sample was heated to 70°C and eventually cooled down to r.t. for 5 min to allow re-folding prior to all measurements. If not indicated otherwise, all experiments were carried out at 25°C.

RNA UV thermal melting

UV thermal melting curves were recorded using a UV-visible spectrophotometer (Cary 100, Agilent former Varian, Switzerland) by measuring the RNA absorbance at 260 nm as a function of temperature. Absorption at 400 nm, which should be constant and close to zero, was used as a control to discard any artifacts from the measurements. RNA samples were prepared with a final concentration of 1 µM in 20 mM HEPES buffer with pH 7.4 (25°C) containing 116 mM of KCl and varying amounts of $MgCl_2$ (0 … 100 mM). to probe the stabilizing effect of divalent cations on both, the kissing loop and the potential KL-TL_{GAAA} docking.

The melting experiments were performed in 10 mm path length quartz cuvettes with 150 µL sample solution, covered with paraffin oil to avoid evaporation. The samples were degassed and pre-heated at 70 °C on a heat block for 5 min, and subsequently cooled down to r.t. After waiting 5 min for temperature stabilization, the melting measurements were started. Temperature ramps were recorded between 15-90°C. A ramp rate of 0.5°C min^{-1} was used and data points were collected every 1 K. As the baseline method was not applicable due to missing baselines at high temperature and two consecutive meltings at higher [Mg^{2+}], T_m's was obtained as local maxima of the first derivative of the absorbance signal $(\mathrm{d}A/\mathrm{d}T)$[16]. [Mg(II)] dependent melting temperatures of the KL- TL_{GAAA} construct (note: the RNA sequence of the KL construct is identical and differs only with regard to the chosen FRET trajectory) are presented in the main text Fig. 6e and in Supplementary Fig. 5.

Ensemble FRET measurements

Fluorescence emission intensities of both fluorophores, the donor (550-620 nm) I_D and the acceptor (640 – 750 nm) I_A were measured for donor excitation (530 nm) and acceptor excitation (630nm) with a fluorescence spectrometer (CARY eclipse, Agilent former Varian, Switzerland). Correction factors for ensemble FRET measurements such as bleed-through (5.2%) from the donor into the acceptor channel and direct excitation (3.0%) of the acceptor upon donor excitation were determined according to standard protocols [17]. Fluorescent background was neglectable. The ensemble FRET efficiency $FRET_{en}$ was then calculated from the fully corrected donor I_D and acceptor I_A intensities according to

$$FRET_{en} = \frac{I_A}{I_A + I_D}\ .$$

The *FRET* proximity ratios of all FRET constructs were plotted against [K^+], [Mg^{2+}] (with a background of 20 mM HEPES buffer with a pH 7.4 (25°C) containing 116 mM KCl, with and without 10 µM Puf6-PUM) for all RNA *in vitro* constructs (Fig. 6c and Supplementary Tables 4-6).

RNA folding and characterization of the thermodynamic stability

According to the observed differences in FRET changes upon addition of mono- and divalent cations for both FRET constructs, the KL and the KL- TL_{GAAA}, probing the KL formation and the docking of the GAAA tetraloop motif to the KL independently, the RNA folding pathway is divided into two subsequent steps $\mathrm{U} \leftrightarrow \mathrm{I} \leftrightarrow \mathrm{F}$, i.e., folding from the unfolded state U to an intermediate state I, thus, the formation of the kissing loop as intermediate state I, and the final docking of the GAAA tetraloop to the KL as folded state F.

RNA folding is usually regarded as hierarchical process, where secondary structures are formed before the RNA eventually collapses in its final three-dimensional fold, its tertiary structure. The building of secondary structure elements (base-paired regions) is associated with the charge compensation of diffusively bound monovalent ions, such as potassium(I) probed herein. In the simplest way, the charge screening can be described as average binding of *n* potassium(I) ions to form the intermediate state I

$$\mathrm{U} + n\,\mathrm{K}^+ \leftrightarrow \mathrm{I} \bullet (\mathrm{K}^+)_n.$$

The associated equilibrium constant is given by

$$K_{\mathrm{eq,K}^+} = \frac{[\mathrm{I}\bullet(\mathrm{K}^+)_n]}{[\mathrm{U}][\mathrm{K}^+]^n},$$

which translates into the well-known Hill equation

$$f_{\mathrm{I}}([\mathrm{K}^+]) = \frac{[\mathrm{K}^+]^n}{K_{\mathrm{eq,K}^+} + [\mathrm{K}^+]^n}\ ,$$

where f_I is the fraction of state I, thus, the KL formed, K_{eq} the associated equilibrium constant, and *n* the Hill coefficient. The experimental measure to observe the folded fraction is the change in $FRET_{\mathrm{en}}([\mathrm{K}^+])$, between a low $FRET_{en,\ min}$ and a high $FRET_{en,\ max}$ value attributed to the transition from the unfolded state U to the folded F or intermediate state I, respectively,

$$FRET_{\mathrm{en}}([\mathrm{K}^+]) = FRET_{en,min} + FRET_{en,max} \frac{[\mathrm{K}^+]^n}{K_{\mathrm{eq,K}^+} + [\mathrm{K}^+]^n} \qquad \text{(SI1)}$$

with

$$f_{\mathrm{I}} = \left(FRET_{\mathrm{en}}([\mathrm{K}^{+}]) - FRET_{\mathrm{en,min}}/FRET_{\mathrm{en,max}}\right)$$

and f_U = 1- f_I as the folded and unfolded fraction of the particular RNA.

Complex formation between a receptor (e.g. kissing loop) and a ligand (GAAA tetraloop motif) is linked to Mg^{2+} binding, where the receptor exists in either unbound (U) or bound (B) form in the absence or presence of Mg^{2+}. The coupling of Mg^{2+} binding and folding has often been described by the following chemical equilibrium

$$\mathrm{U} + n\,\mathrm{Mg}^{2+} \leftrightarrow \mathrm{B} \bullet \left(\mathrm{Mg}^{2+}\right)_{n},$$

where *m* refers to the number of discrete Mg^{2+} ions associated with the bound state. We associate the Mg^{2+} unbound state with the intermediate state I and the Mg^{2+} bound state with the folded state F

$$\mathrm{I} + n\,\mathrm{Mg}^{2+} \leftrightarrow \mathrm{F} \bullet \left(\mathrm{Mg}^{2+}\right)_{n}.$$

The associated equilibrium constant or apparent dissociation constant is given by

$$K_{\mathrm{eq,Mg}^{2+}} = \frac{\left[\mathrm{F} \bullet \left(\mathrm{Mg}^{2+}\right)_{n}\right]}{[\mathrm{I}][\mathrm{Mg}^{2+}]^{n}}$$

and translates with the folded fraction of the F state

$$f_{\mathrm{F}} = \left(FRET_{en}\left(\left[\mathrm{Mg}^{2+}\right]\right) - FRET_{\mathrm{en,min}}\right)/FRET_{\mathrm{en,max}}$$

into the well-known Hill equation

$$FRET_{\mathrm{en}}([\mathrm{Mg}^{2+}]) = FRET_{\mathrm{en,min}} + FRET_{\mathrm{en,max}} \frac{[\mathrm{Mg}^{2+}]^{n}}{K_{\mathrm{eq,Mg}^{2+}} + [\mathrm{Mg}^{2+}]^{n}}, \qquad \text{(SI2)}$$

where $FRET_{\mathrm{en}}\left(\left[\mathrm{Mg}^{2+}\right]\right)$ is the fraction of docked GAAA tetraloop to the KL/tetraloop receptor with bound $\left[\mathrm{Mg}^{2+}\right]$. Fitting results are summarized in Supplementary Tables 4-6.

The Gibbs free energy corresponding to the midpoint concentration of a respective transition between two folded states, here the K^{+} dependent KL formation

$$\Delta_r G^{\circ\,\mathrm{K}^{+}}_{\mathrm{IU}} = \Delta G_{\mathrm{KL}}$$

and the Mg^{2+} dependent KL/TLR-TL_{GAAA} docking

$$\Delta_r G^{\circ\,\mathrm{Mg}^{2+}}_{\mathrm{FI}} = \Delta G_{\mathrm{KL-TL}_{GAAA}}$$

at standard conditions (25°C, 100kPA) is calculated according to

$$\Delta_r G^\circ = \Delta G_{25°C} = -RT \ln(K_{eq}). \quad \text{(SI3)}$$

Results are summarized in Supplementary Tables 4-6.

Mg^{2+} dependent UV thermal melting experiments report on the change in stability due to magnesium (II) binding. Similar to the FRET folding studies, we describe the thermal melting experiments with a modified Hill equation

$$T([\mathrm{Mg}^{2+}]) = T_{min} + T_{max} \frac{[\mathrm{Mg}^{2+}]^{\mathrm{n}}}{K_{\mathrm{eq}}+[\mathrm{Mg}^{2+}]^{\mathrm{n}}}. \quad \text{(SI4)}$$

Results are summarized in Supplementary Table 7.

Determination of affinities by fluorescence anisotropy

Fluorescence anisotropy data was measured in non-binding 96-well plates (Greiner Bio-One) using a Tecan Safire II plate reader (Tecan Group Ltd.) equipped with a fluorescence polarization module. A constant amount of 2.5 nM Cy5-labeled H68- or H68-H88-H22-RNA (IBA Lifesciences) was titrated with increasing concentrations ranging from 0.16 nM to 20 µM Puf6-PUM (161–656 aa), which resulted in a total of 24 dilution points. The excitation wavelength was set to 635 nm and the emission was measured at 670 nm using a bandwidth of 10 nm. Each titration experiment was measured in four replicates and the averages were normalized with the anisotropy corresponding to the lowest Puf6 concentration and were fit to

$$\mathrm{Y}(\mathrm{K_D}, \mathrm{P}, \mathrm{R}) = \frac{m\left(-\mathrm{K_D}-\mathrm{P}-\mathrm{R}+\sqrt{(\mathrm{K_D}+\mathrm{P}+\mathrm{R})^2-4\mathrm{PR}}\right)}{-2\mathrm{R}},$$

where Y is the bound fraction of Cy5-labeled RNA, K_D is the dissociation constant, P is the Puf6 concentration, R is the RNA concentration and m is a scaling factor to convert concentrations into anisotropy.

DDA data analysis

The raw DDA files were converted to mzXML using the qtofpeakpicker component of msconvert (Proteowizzard v 3.0.9987) then to mgf using MzXML2Search (TPP v4.7 rev 0). The converted files searched with Comet (2016.01 rev. 2) or Mascot (v 2.5.1) using the SGD yeast database (released 13.01.2015, containing 6’713 proteins plus one concatenated iRT protein entry and as many decoy entries generated by pseudo-reversing the tryptic peptide sequences). The search parameters were as follows: +/- 25 ppm tolerance for MS1 and MS2; fixed cysteine carbamidomethylation and variable methionine oxidation; semi-tryptic and two missed cleavage allowed. The comet and mascot search results were processed using

peptideProphet [18] and aggregated using iProphet [19] (TPP v4.7 rev 0). The search results were filtered for an iProphet cutoff of 0.98933, corresponding to a 1% protein false discovery rate estimated by MAYU [20]. The search results contained 29'657 peptides matching to a maximal set of 3229 expanded proteins. The consensus spectral library was generated using spectrast [21] and the assay library thereof was exported using the spectrast2tsv.py script [22] with the following parameters: 6 highest intensity fragments (of charge 1+ or 2+) per peptide, within the mass range 350-2000 m/z and excluding the fragments within the precursor isolation window of the corresponding swath. The final library contained assays for 26'702 peptide precursors (thereof 24'127 proteotypic assay precursors covering 2758 unique proteins). The assay library was exported to TraML with shuffle decoys appended as described in [22].

SWATH-MS data analyses

The SWATH MS data was extracted with the above-mentioned assay library through the iPortal interface [23] with openSWATH [24] (openMS 2.1.0), pyProphet [25] and TRIC alignment [26] using the same parameters as described in [27]. From the SWATH extraction output results, the identified assays/precursors were further filtered for m-score below 0.00013 (corresponding to a protein FDR of 1%) resulting in 21154 precursor identifications mapping to 2821 proteins. The non-proteotypic peptides were filtered out, as well as the assays with CV within replicates beyond 30% in more than two conditions (Supplementary Figs. 1a-c). The precursor intensity values were log2-transformed and were mean-transformed: first the median log2 intensity value was calculated for each run and subtracted run-wise to each value (Supplementary Fig. 1d); then the global mean across all the samples (run-wise) was added to all runs (Supplementary Fig. 1e). Single hit proteins (identified with a single peptide only) were filtered out. Missing values were put in per run using 80% of the minimal effective (not log) assay value found in that run. Protein abundance was calculated by summing up all the effective assay/precursor values for a given protein. The proteins were tested for regulation across the four temperature triplicate conditions by one-way ANOVA and the p-value was adjusted using the FDR method from the R p.adjust function (Supplementary Fig. 1g, h). Overall, 478 proteins were found significantly regulated with adjusted p-values below 0.01 and a log2 fold change above log2(1.5) from that of the overall protein mean (Supplementary Data 1). The log2 intensities of those regulated proteins across the 12 runs were then standardized and processed for soft/fuzzy c-means clustering using the R (v 3.6.3) Mfuzz package (v 2.44) [28] (Supplementary Fig.1g, i). The nine most significant clusters as suggested by the Dmin and overlap functions of mFuzz (Supplementary Fig. 1j and k) (embedding 429 of the significantly regulated proteins) are provided in the Supplementary Data 1.

Supplementary Table 3. Sequences and structural predictions of the fluorescently labelled RNA constructs for ensemble FRET and fluorescence anisotropy measurements

construct	Knowledge-based structure prediction*	RNAfold
KL	UGAAGAAAUUCAaaaaaaaaGCUCGGAAUUUG<br>AGCaaaaaaaaaaaaCGGUGGUAAAUUCCAUC<br>G<br>((((....)))).........((((.[[[[[[)<br>)))............(((((..]]]]]]))))<br>)[1]	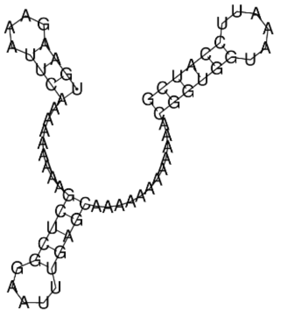
KL-TL$_{GAAA}$**	UGAAGAAAUUCAaaaaaaaaGCUCGGAAUUUG<br>AGCaaaaaaaaaaaaCGGUGGUAAAUUCCAUC<br>G<br>((((....)))).........((((.[[[[[[)<br>)))............(((((..]]]]]]))))<br>)[1]	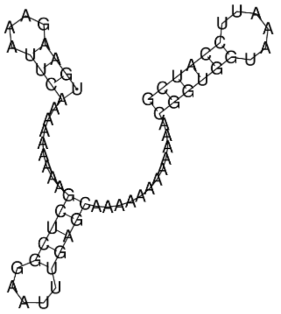
TLR-TL$_{GAAA}$	UGAAGAAAUUCAAAAAAAAACGUGUCGUCCUA<br>AGUCGGCAUUCGUGCCGAUAUGGACGACACG<br>((((....)))).........((((((((((..<br>.(((((((....)))))))..))))))))))	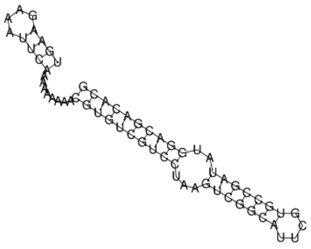
H68**	UGAAGAAAUUCA<br>((((....))))	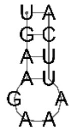

*sCy5 in red, sCy3 in green
**constructs used for fluorescence anisotropy measurements

[1]Bracket () for based-pairs within one helix and [] for base-pairs between different helices, thus forming a pseudoknot, i.e. a kissing loop.

Supplementary Table 4. Fitting results for the kissing loop (KL) construct according to Eqn. (SI1 – SI3).

Condition	$FRET_{max}$	K_A	n	$K_{eq} = (K_A)^n$	$\Delta_r G°$(kJ mol^{-1})
Water only	0.05±0.00(3)*	-	-	-	-
K(I) total	0.40±0.04	16±1 mM	1.1±0.1	21±1 mM	9.6
Mg(II)	0.44±0.01	1.3±0.9 mM	1.1±0.4	1.3±0.9 mM	16.4
Mg(II) + Puf6-PUM	0.53	undefined	undefined	-	-

* experimental value, no fitting result # ligand concentration producing half occupation

Supplementary Table 5. Fitting results for the KL-TL$_{GAAA}$ construct according to Eqn. (SI1 – SI3)

Condition	$FRET_{max}$	K_A#	n	$K_{eq} = (K_A)^n$	$\Delta_r G°$(kJ mol^{-1})
Water only	0.03±0.00(2)*	-	-	-	-
K(I) total	0.25±0.04	17±8 mM	0.9±0.4	13±8 mM	10.8
Mg(II)	0.42±0.01	2.2±0.6 mM	1.0±0.2	2.2±0.6 mM	15.2
Mg(II) + Puf6-PUM	0.49*	undefined	undefined	-	-

* experimental value, no fitting result # ligand concentration producing half occupation

Supplementary Table 6. Fitting results for the classical TL receptor, TLR-TL$_{GAAA}$ construct according to Eqn. (SI1 – SI3)

Condition	$FRET_{max}$	K_A#	n	$K_{eq} = (K_A)^n$	$\Delta_r G°$(kJ mol^{-1})
Water only	0.05±0.00(3)*	-	-		
K(I) total	0.28±0.25	140±565 mM	0.46±0.28	9.7± mM	11.8
Mg(II)	0.39±0.01	3.2±0.4 mM	1.0±0.1	3.2±0.4 mM	9.6
Mg(II) + Puf6-PUM	0.44*	undefined	undefined	-	-

* experimental value, no fitting result # ligand concentration producing half occupation

Supplementary Table 7. Thermodynamic characterization and transition midpoints according to Eq. (SI4) of the Mg^{2+}-dependent melting experiments probing the KL formation and the KL-TL$_{GAAA}$ docking.

Construct	KL	TL$_{GAAA}$-KL
$K_{eq.Mg(II)}$	2.8±0.6 mM	<5 mM (not determined)
$T_{M, no Mg(II)}$	66°C	<25°C (not observed)
T_M @ K_{eq}	71°C	About 30°C
$T_{M, 100mM Mg(II)}$	77°C	35°C

Supplementary References

1. SenGupta, D. J. *et al.* A three-hybrid system to detect RNA-protein interactions in vivo. *Proc. Natl. Acad. Sci. U. S. A.* **93**, 8496–8501 (1996).

2. Altvater, M. *et al.* Targeted proteomics reveals compositional dynamics of 60S pre-ribosomes after nuclear export. *Mol. Syst. Biol.* **8**, 628 (2012).

3. Altvater, M., Schütz, S., Chang, Y. & Panse, V. G. Dissecting ribosome assembly and transport in budding yeast. in *Methods in Cell Biology* vol. 122 437–461 (Academic Press Inc., 2014).

4. Bassler, J., Kallas, M. & Hurt, E. The Nug1 GTPase reveals an N-terminal RNA-binding domain that is essential for association with 60 S pre-ribosomal particles. *J. Biol. Chem.* **281**, 24737–24744 (2006).

5. Wu, S. *et al.* Diverse roles of assembly factors revealed by structures of late nuclear pre-60S ribosomes. *Nature* **534**, 133–137 (2016).

6. Sanghai, Z. A. *et al.* Modular assembly of the nucleolar pre-60S ribosomal subunit. *Nature* **556**, 126–129 (2018).

7. Zhou, Y., Musalgaonkar, S., Johnson, A. W. & Taylor, D. W. Tightly-orchestrated rearrangements govern catalytic center assembly of the ribosome. *Nat. Commun.* **10**, 1–11 (2019).

8. Kemmler, S., Occhipinti, L., Veisu, M. & Panse, V. G. Yvh1 is required for a late maturation step in the 60S biogenesis pathway. *J. Cell Biol.* **186**, 863–880 (2009).

9. Lo, K. Y., Li, Z., Wang, F., Marcotte, E. M. & Johnson, A. W. Ribosome stalk assembly requires the dual-specificity phosphatase Yvh1 for the exchange of Mrt4 with P0. *J. Cell Biol.* **186**, 849–862 (2009).

10. Lau, B. *et al.* Structure of the Maturing 90S Pre-ribosome in Association with the RNA Exosome. *Mol. Cell* **81**, (2020).

11. Schuller, J. M., Falk, S., Fromm, L., Hurt, E. & Conti, E. Structure of the nuclear exosome captured on a maturing preribosome. *Science (80-.).* **360**, 219–222 (2018).

12. Steffen, F. D., Sigel, R. K. O. & Börner, R. An atomistic view on carbocyanine photophysics in the realm of RNA. *Phys. Chem. Chem. Phys.* **18**, 29045–29055 (2016).

13. Pyle, A. M. Metal ions in the structure and function of RNA. *Journal of Biological Inorganic Chemistry* vol. 7 679–690 (2002).

14. Antczak, M. *et al.* New functionality of RNAComposer: An application to shape the axis of miR160 precursor structure. *Acta Biochim. Pol.* **63**, 737–744 (2016).

15. Erichson, F., Steffen, F. D. & Börner, R. FRET-assisted structural model of the GAAA RNA tetraloop receptor,. in *26. Interdisziplinäre Wissenschaftliche Konferenz Mittweida, Scientific Reports 2021, ISSN 1437-7624* 230–233 (2021). doi:https://doi.org/10.48446/opus-12283.

16. Mergny, J. L. & Lacroix, L. Analysis of Thermal Melting Curves. *Oligonucleotides* vol. 13 515–537 (2003).

17. Börner, R. *et al.* Simulations of camera-based single-molecule fluorescence experiments. *PLoS One* **13**, e0195277 (2018).

18. Keller, A., Nesvizhskii, A. I., Kolker, E. & Aebersold, R. Empirical statistical model to estimate the accuracy of peptide identifications made by MS/MS and database search. *Anal. Chem.* **74**, 5383–5392 (2002).

19. Shteynberg, D. *et al.* iProphet: Multi-level Integrative Analysis of Shotgun Proteomic Data Improves Peptide and Protein Identification Rates and Error Estimates. *Mol. Cell. Proteomics* **10**, M111.007690 (2011).

20. Reiter, L. *et al.* Protein identification false discovery rates for very large proteomics data sets generated by tandem mass spectrometry. *Mol. Cell. Proteomics* **8**, 2405–2417 (2009).

21. Lam, H. *et al.* Building consensus spectral libraries for peptide identification in proteomics. *Nat. Methods* **5**, 873–875 (2008).

22. Schubert, O. T. *et al.* Building high-quality assay libraries for targeted analysis of SWATH MS data. *Nat. Protoc.* **10**, 426–441 (2015).

23. Kunszt, P. *et al.* iPortal: the swiss grid proteomics portal: Requirements and

new features based on experience and usability considerations. *Concurr. Comput. Pract. Exp.* **27**, 433–445 (2015).

24. Röst, H. L. *et al.* OpenSWATH enables automated, targeted analysis of data-independent acquisition MS data. *Nature Biotechnology* vol. 32 219–223 (2014).

25. Teleman, J. *et al.* DIANA—algorithmic improvements for analysis of data-independent acquisition MS data. *Bioinformatics* **31**, 555–562 (2015).

26. Röst, H. L. *et al.* TRIC: An automated alignment strategy for reproducible protein quantification in targeted proteomics. *Nat. Methods* **13**, 777–783 (2016).

27. Navarro, P. *et al.* A multicenter study benchmarks software tools for label-free proteome quantification. *Nat. Biotechnol.* **34**, 1130–1136 (2016).

28. Kumar, L. & Futschik, M. E. Mfuzz: A software package for soft clustering of microarray data. *Bioinformation* **2**, 5–7 (2007).
