# Supplementary material for: Puf6 primes 60S pre-ribosome nuclear export at low temperature
Source: Nat Commun. 2021 Aug 4;12:4696. doi: 10.1038/s41467-021-24964-2 (PMC8338941; doi:10.1038/s41467-021-24964-2)
Supplement: Supplementary file 3 — Description of Additional Supplementary Files [file 41467_2021_24964_MOESM3_ESM.pdf]

Description of Additional Supplementary Files

File Name: Supplementary Data 1
Description: Protein abundance values of detected proteins from SWATHMS analysis and DAVID annotation clustering analysis showing members of each of the nine-protein profile cluster and their GO term enrichment.

File Name: Supplementary Data 2
Description: Complete list of individual protein-protein crosslinks from XLMS analysis
