# Supplementary material for: Puf6 primes 60S pre-ribosome nuclear export at low temperature
Source: Nat Commun. 2021 Aug 4;12:4696. doi: 10.1038/s41467-021-24964-2 (PMC8338941; doi:10.1038/s41467-021-24964-2)
Supplement: Supplementary file 6 — Reporting Summary [file 41467_2021_24964_MOESM6_ESM.pdf]

nature portfolio

Corresponding author(s): Vikram Govind Panse

Last updated by author(s): Jul 6, 2021

Reporting Summary

Nature Portfolio wishes to improve the reproducibility of the work that we publish. This form provides structure for consistency and transparency in reporting. For further information on Nature Portfolio policies, see our Editorial Policies and the Editorial Policy Checklist.

Statistics

For all statistical analyses, confirm that the following items are present in the figure legend, table legend, main text, or Methods section.

n/a	Confirmed	
☐	☒	The exact sample size (*n*) for each experimental group/condition, given as a discrete number and unit of measurement
☐	☒	A statement on whether measurements were taken from distinct samples or whether the same sample was measured repeatedly
☐	☒	The statistical test(s) used AND whether they are one- or two-sided<br>*Only common tests should be described solely by name; describe more complex techniques in the Methods section.*
☒	☐	A description of all covariates tested
☒	☐	A description of any assumptions or corrections, such as tests of normality and adjustment for multiple comparisons
☒	☐	A full description of the statistical parameters including central tendency (e.g. means) or other basic estimates (e.g. regression coefficient) AND variation (e.g. standard deviation) or associated estimates of uncertainty (e.g. confidence intervals)
☐	☒	For null hypothesis testing, the test statistic (e.g. *F*, *t*, *r*) with confidence intervals, effect sizes, degrees of freedom and *P* value noted<br>*Give P values as exact values whenever suitable.*
☒	☐	For Bayesian analysis, information on the choice of priors and Markov chain Monte Carlo settings
☒	☐	For hierarchical and complex designs, identification of the appropriate level for tests and full reporting of outcomes
☒	☐	Estimates of effect sizes (e.g. Cohen's *d*, Pearson's *r*), indicating how they were calculated

Our web collection on statistics for biologists contains articles on many of the points above.

Software and code

Policy information about availability of computer code

Data collection	Details are described in Methods and Supplementary information in Sections: CRAC analysis, SWATH-MS sample preparation for LC-MS/MS analyses; Cross-linking MS; Purification of pre-ribosomal particles; TAP purification; Fluorescence microscopy and heterokaryon assay
Data analysis	SWATH-MS:<br><br>DDA data format conversion by msconvert (Proteowizzard v 3.0.9987) and MzXML2Search (TPP v4.7 rev 0)<br><br>Converted data searched with Comet (2016.01 rev. 2) and Mascot (v 2.5.1) tools using SGD database (released 13.01.2015)<br><br>Data aggregation using iProphet (TPP v4.7 rev 0) tool<br><br>Spectral library generated by SpectraST software (v 5.0, TPP v4.7) and exported using spectrast2tsv.py script (part of msproteomicstools version 0.4.3)<br><br>SWATH-MS data extracted through the iPortal interface with openSWATH (openMS 2.1.0), pyProphet (0.22) and TRIC alignment (part of msproteomicstools version 0.4.3).<br><br>Clustering analysis: R (v 3.6.3) using the Mfuzz package (v 2.44)<br><br>XL-MS: Data analysis was performed using xQuest/xProphet (v 2.1.3).<br><br>CRAC analysis: CRAC analysis: pyCRAC-1.42 software suite

(https://git.ecdf.ed.ac.uk/sgrannem/pycrac) and mapped to the yeast genomic reference sequence Saccharomyces_cerevisiae. R64-1-1.75 using NovoAlign-2.07 (http://www.novocraft.com). Plots of reads aligned to the 35S rRNA sequence were made with the pyPileup module of pyCRAC

Secondary RNA structure prediction server: ViennaRNA

Microscopy images were acquired by Openlab software and processed using ImageJ (v2)

Figures of molecular structures created by PyMOL (2.2.0)

Sequence alignment: generated by MAFFT software (v7), visualized with Jalview (v2.11.1.4)

Protein structural modelling: Phyre2 server (http://www.sbg.bio.ic.ac.uk/phyre)

RNA 3D structure prediction: RNAcomposer

FRET measurements: data analysis and plots were made using OriginPro 2020 (64-bit) SR1 (9.7.0.188)

For manuscripts utilizing custom algorithms or software that are central to the research but not yet described in published literature, software must be made available to editors and reviewers. We strongly encourage code deposition in a community repository (e.g. GitHub). See the Nature Portfolio guidelines for submitting code & software for further information.

Data

Policy information about availability of data

All manuscripts must include a data availability statement. This statement should provide the following information, where applicable:

- Accession codes, unique identifiers, or web links for publicly available datasets
- A description of any restrictions on data availability
- For clinical datasets or third party data, please ensure that the statement adheres to our policy

All datasets generated in the study are deposited in repositories with unique identifiers and are made public: SWATH-MS and XL-MS datasets can be found at the ProteomeXchange Consortium via the PRIDE partner repository with unique identifiers PXD016320 and PXD024131, respectively. The CRAC-dataset has been deposited into the Gene Expression Omnibus database Accession No. GSE174587. All source data are provided as a source file along with the manuscript.

Field-specific reporting

Please select the one below that is the best fit for your research. If you are not sure, read the appropriate sections before making your selection.

[x] Life sciences [ ] Behavioural & social sciences [ ] Ecological, evolutionary & environmental sciences

For a reference copy of the document with all sections, see nature.com/documents/nr-reporting-summary-flat.pdf

Life sciences study design

All studies must disclose on these points even when the disclosure is negative.

Sample size	Samples for mass spectrometry were prepared, processed and acquired in biological triplicates. All biochemistry and cell-biological studies were performed with defined components (Puf6/protein and RNA constructs), hence in these instances sample size is not applicable.
Data exclusions	All the mass spectrometry, biochemical, bio-physical and cell-biological analyses are presented. No data was excluded.
Replication	All experiments were performed at least 2-3 times; where applicable p values and appropriate errors are provided and stated in the Figure Legend/Text.
Randomization	Mass spectrometry: Due to the small sample size (12 samples), the samples could all be processed and measured in a single batch. No batch effect was observed (Supplementary Fig. 1F)<br>Biochemical and cell-biological studies were carried out with defined components (Puf6/protein and RNA), hence randomization is not applicable.
Blinding	All mass spectrometry biochemical and biophysical employ defined components (Puf6/protein, RNA) and cell-biological studies employed defined yeast cells expressing GFP-fusions, hence blinding is not relevant.

Reporting for specific materials, systems and methods

We require information from authors about some types of materials, experimental systems and methods used in many studies. Here, indicate whether each material, system or method listed is relevant to your study. If you are not sure if a list item applies to your research, read the appropriate section before selecting a response.

Materials & experimental systems

n/a	Involved in the study
☐	☒ Antibodies
☒	☐ Eukaryotic cell lines
☒	☐ Palaeontology and archaeology
☒	☐ Animals and other organisms
☒	☐ Human research participants
☒	☐ Clinical data
☒	☐ Dual use research of concern

Methods

n/a	Involved in the study
☒	☐ ChIP-seq
☒	☐ Flow cytometry
☒	☐ MRI-based neuroimaging

Antibodies

Antibodies used	Primary antibodies were used in the following dilutions: α-Puf6 (1:2000; this study), α-Nog2 (1:1000, this study), α-Nmd3 (1:1000, A. Johnson, University of Texas, Austin, USA), α-Sda1 (1:1000, this study), α-Nog2 (1:500, this study), α-TAP (CBP) (1:1000, Merck AG, Darmstadt, Germany #07-482), α-uL29 (1:2000; V. Panse, University of Zurich, Switzerland), α-Arc1 (1:4000, E Hurt, University of Heidelberg, Heidelberg, Germany). The secondary HRP-conjugated α rabbit antibody (Cat No. I5006; Sigma-Aldrich,St Louis, MO, USA) was used at 1:2000 dilution.
Validation	anti-Puf6, anti-Sda1, u-L29 and anti-Nog2 antibodies were raised against recombinant yeast proteins, respectively and validated by performing Western analyses of yeast lysates and recombinant proteins. All antibodies used in this study have been validated by numerous research groups including ours (Altvater et al., 2012 Mol. Sys. Biol.-DOI: 10.1038/msb.2012.63; Klingauf-Nerurkar et al., 2020 eLife-DOI: 10.7554/eLife.52474). All antibodies are polyconal raised in rabbits using recombinant yeast proteins.
